# Electrically-evoked responses for retinal prostheses are differentially altered depending on ganglion cell types in outer retinal neurodegeneration caused by *Crb1* gene mutation

**DOI:** 10.3389/fncel.2023.1115703

**Published:** 2023-02-06

**Authors:** Hyeonhee Roh, Yanjinsuren Otgondemberel, Jeonghyeon Eom, Daniel Kim, Maesoon Im

**Affiliations:** ^1^Brain Science Institute, Korea Institute of Science and Technology, Seoul, Republic of Korea; ^2^School of Electrical Engineering, Korea University, Seoul, Republic of Korea; ^3^School of Electrical Engineering, Kookmin University, Seoul, Republic of Korea; ^4^Department of Biomedical Sciences, Seoul National University College of Medicine, Seoul, Republic of Korea; ^5^Division of Bio-Medical Science & Technology, KIST School, University of Science and Technology, Seoul, Republic of Korea

**Keywords:** retinitis pigmentosa, retinal degeneration, artificial vision, retinal prosthesis, electrical stimulation

## Abstract

**Background:**

Microelectronic prostheses for artificial vision stimulate neurons surviving outer retinal neurodegeneration such as retinitis pigmentosa (RP). Yet, the quality of prosthetic vision substantially varies across subjects, maybe due to different levels of retinal degeneration and/or distinct genotypes. Although the RP genotypes are remarkably diverse, prosthetic studies have primarily used retinal degeneration (*rd*) 1 and 10 mice, which both have *Pde6b* gene mutation. Here, we report the electric responses arising in retinal ganglion cells (RGCs) of the *rd8* mouse model which has *Crb1* mutation.

**Methods:**

We first investigated age-dependent histological changes of wild-type (*wt*), *rd8*, and *rd10* mice retinas by H&E staining. Then, we used cell-attached patch clamping to record spiking responses of ON, OFF and direction selective (DS) types of RGCs to a 4-ms-long electric pulse. The electric responses of *rd8* RGCs were analyzed in comparison with those of *wt* RGCs in terms of individual RGC spiking patterns, populational characteristics, and spiking consistency across trials.

**Results:**

In the histological examination, the *rd8* mice showed partial retinal foldings, but the outer nuclear layer thicknesses remained comparable to those of the *wt* mice, indicating the early-stage of RP. Although spiking patterns of each RGC type seemed similar to those of the *wt* retinas, correlation levels between electric vs. light response features were different across the two mouse models. For example, in comparisons between light vs. electric response magnitudes, ON/OFF RGCs of the *rd8* mice showed the same/opposite correlation polarity with those of *wt* mice, respectively. Also, the electric response spike counts of DS RGCs in the *rd8* retinas showed a positive correlation with their direction selectivity indices (*r* = 0.40), while those of the *wt* retinas were negatively correlated (*r* = −0.90). Lastly, the spiking timing consistencies of late responses were largely decreased in both ON and OFF RGCs in the *rd8* than the *wt* retinas, whereas no significant difference was found across DS RGCs of the two models.

**Conclusion:**

Our results indicate the electric response features are altered depending on RGC types even from the early-stage RP caused by *Crb1* mutation. Given the various degeneration patterns depending on mutation genes, our study suggests the importance of both genotype- and RGC type-dependent analyses for retinal prosthetic research.

## 1. Introduction

**FIGURE 1:**
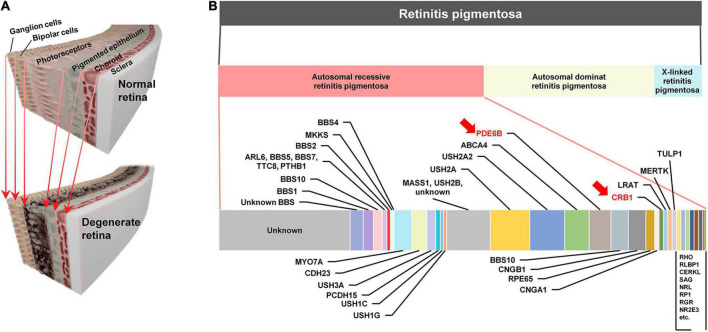
**(A)** Schematic illustrations of the healthy retina (top) and the retina damaged by outer retinal degenerative diseases such as retinitis pigmentosa (bottom). As the retina degenerates, the photoreceptors are primarily damaged. **(B)** Spectrum of retinitis pigmentosa (RP) (second row) RP has three major types autosomal recessive retinitis pigmentosa (ARRP; 50–60%), autosomal dominant retinitis pigmentosa (ADRP; 30–40%), and X-linked RP (5–15%) (last row). Estimated relative contributions of various genes causing ARRP in human patients. The estimated proportions are from a previous work (Hartong et al., 2006). Among the causal genes listed in this horizontal bar chart, *PDE6B* and *CRB1* are marked with red arrows, which are corresponding to murine mutation genes (i.e., *Pde6b* and *Crb1*) of retinal degeneration (*rd*) 10 and *rd8* mouse models, respectively.

Outer retinal degenerative diseases such as age-related macular degeneration (AMD) and retinitis pigmentosa (RP) cause loss of photoreceptors (Figure 1A), subsequent remodeling of retinal neural circuits, and profound visual impairment (Marc and Jones, 2003; Marc et al., 2003; Hartong et al., 2006; Jager et al., 2008). In the past two decades, various microelectronic prosthetic systems have demonstrated electrical stimulation of surviving neurons can elicit spiking activities in retinal ganglion cells (RGCs), offering artificial visual percepts in blind individuals (Humayun et al., 1996; Rizzo et al., 2003; Zrenner et al., 2011). Also, several clinical trials showed quite promising outcomes of Argus II (Second Sight), Alpha IMS/AMS (Retina Implant AG), and PRIMA (Pixium Vision), making those retinal prostheses commercialized in the past or near commercialization (Humayun et al., 1996; Ahuja et al., 2011; Zrenner et al., 2011; da Cruz et al., 2013; Stingl et al., 2015; Palanker et al., 2020, 2022). However, the best quality of electrically-evoked prosthetic vision (20/460) (Palanker et al., 2020) has reached yet to neither the level of independent walks without guide dog/cane nor the level of legal blindness (20/200). Moreover, prosthetic users who suffered from RP showed considerably different levels of restored vision (Stingl et al., 2015), hindering the wide use of retinal prostheses. For instance, some retinal prosthetic users were able to recognize/localize testing objects while others were unable to perceive any artificial visual sensation (Stingl et al., 2015).

The remarkable variety of disease genotypes can be one of the critical factors that may have caused the substantial performance variation. For example, inheritance of RP can be classified into three major groups (second row of Figure 1B): autosomal recessive (50–60%), autosomal dominant (30–40%), and X-linked (5–15%) RPs (Hartong et al., 2006). In addition to the highest proportion, the autosomal recessive RP (ARRP) has remarkably heterogeneous genotypes (bottom row of Figure 1B), which are known to, along with other factors (e.g., environment, gender, and epigenetics), result in the different levels/patterns of retinal degeneration in each individual (Chang et al., 2011). Similar with RP, AMD genotypes are also variegated, which include single nucleotide polymorphisms (SNPs) in vascular endothelial growth factor (*VEGF*), and pigment epithelium-derived factor (*PEDF*), complement factor H (*CFH*), and high-temperature requirement factor A1 (*HTRA1*) genes (Mori et al., 2010; Black and Clark, 2016; Battu et al., 2022). Each genotype disrupts distinct pathways and/or functions of the retina and expresses unique phenotypes (Berger et al., 2010; Verbakel et al., 2018), raising a possibility that electrically-evoked spiking responses (hereafter referred to as electric responses) of RGCs are significantly different depending on the genotype. Therefore, comprehensive neurophysiological understandings of electric responses arising in each genotype are necessary for advanced vision restoration for both RP and AMD patients.

In previous retinal prosthetic researches using RP animal models, retinal degeneration 1 (*rd1*) and 10 (*rd10*) mice have been widely used, which are the well-established mouse models of the ARRP (Chang et al., 2002; Gargini et al., 2007). Those two models have a *Pde6b* genetic mutation in common, which is responsible for encoding β-subunit of cyclic guanosine monophosphate (cGMP)-specific phosphodiesterase (PDE). It has been known that cGMP PDE plays a crucial role in the phototransduction of photoreceptors as an important enzyme for regulating the level of cGMP, which is an intracellular signaling molecule known as a second messenger (Chang et al., 2002). The difference between the two models is that the *rd10* model mimics human RP better in terms of its slower disease progression than the *rd1* model (Gargini et al., 2007). However, given the fact that the ARRP has numerous different genotypes (Figure 1B; Chang et al., 2002; Hartong et al., 2006; Bravo-Gil et al., 2017; Verbakel et al., 2018), it is essential to examine other genotypes for successful clinical outcomes of retinal prostheses. For example, the mutation of *Crb1* gene is another genotype of the ARRP, which is currently available as *rd8* mouse model (see Table 1 for comparisons across *rd1*, *rd10*, and *rd8* mice) (Mehalow et al., 2003). The *Crb1* gene is strongly associated with epithelial polarity by its emplacement proximal to the adhesion junction in the outer limiting membrane (OLM) (Mehalow et al., 2003; Aredo et al., 2015). Accordingly, retinal foldings and/or disassembled retinal layers were observed in the mouse models carrying *Crb1* mutation (Mehalow et al., 2003; van de Pavert et al., 2004; van de Pavert et al., 2007). Similarly, patients with *CRB1* mutations also showed abnormal laminar structures or retinal telangiectasia with exudation (Jacobson et al., 2003; Bujakowska et al., 2015). Despite of its availability and clinical importance, *rd8* mice have not been well studied in both visual neuroscience and retinal prosthetics.

**TABLE 1:** Qualitative comparisons of major characteristics across *rd1*, *rd10* and *rd8* mouse RP models (Hawes et al., 2000; Chang et al., 2002; Mehalow et al., 2003; Hartong et al., 2006).

	*rd1*	*rd10*	*rd8*
Mutation	*Pde6b* (Nonsense)	*Pde6b* (Missense)	*Crb1*
Phenotype	Vessel attenuation Pigment patch in the fundus	Sclerotic retinal vessels	Large white deposits Retinal folding corresponds to white spot
Chromosome (mouse)	Chr.5	Chr.5	Chr.1
Chromosome (human)	Chr.4p16	Chr.4p16	Chr.1q25
Retinal ONL loss by (month)	1	2	30
Recordable ERG responses	PD14–PD16	PD14–PD28	PD14–PD365
Prevalence (in autosomal recessive population)	4–5% 1%			

Ages of animals showing recordable electroretinogram (ERG) responses are in postnatal days (PD). ONL: Outer Nuclear Layer.

In the present work, we characterized electric responses arising in three distinct physiological types of RGCs in the *rd8* mouse model (e.g., ON, OFF, and ON-OFF direction-selective RGCs). We also correlated electric responses of RGCs with their own visually-evoked responses and compared the correlations with those of wild-type (*wt*) RGCs. Lastly, we examined the spiking consistencies across repeats of electric stimuli in each type. By comparing the response features of those three RGC types across diverse genotypes and healthy animals (e.g., *rd8* and *wt* mice in the present work, *rd1/10* and *wt* mice in the earlier studies) translational differences of microelectronic prostheses may be estimated, which would be applicable to patient sxelection.

## 2. Materials and methods

### 2.1. Animals

The animal experiment protocols were approved by the Institutional Animal Care and Use Committee of the KIST (KIST-2020-156, KIST-2021-09-105, KIST-5088-2022-05-076). Wild-type (*wt*; C57BL/6J strain) and retinal degeneration 8 (*rd8*; C57BL/6N strain) mice were purchased from Daehan BioLink (Eumseong, South Korea) and Young Bio (Seongnam, South Korea), respectively. These two strains were used for electrophysiological recordings and histological analyses. For histological comparison only, *rd10* (B6.CXB1-*Pde6b^rd10^*/J) mice were also used. The first breeding pairs of *rd10* mice were purchased from Jackson Lab (Bar Harbor, ME, USA), and then its colony has been maintained in the KIST animal facility. All mice were anesthetized via inhalation of vaporizing isoflurane and euthanized by cervical dislocation.

### 2.2. Histological analysis

After euthanasia, a mouse eyeball was fixed in David’s fixative and 10% neutral buffered formalin (NBF) solution (GD Chem, Eumseong, South Korea). The eyeball was washed with tap water and embedded in paraffin. The prepared sample was sectioned to be 4 μm in thickness using a rotary microtome (Shandon Finesse ME, Thermo Fisher Scientific, Waltham, MA, USA). The sectioned samples were mounted on each slide glass and dried on a slide warmer (C-SL, Changshin Science, Seoul, South Korea). After that, the slide glass with the retinal tissue was placed into an oven at 58–60°C to increase attachment between the tissue and the slide glass. Lastly, the prepared samples were stained with hematoxylin and eosin (H&E). Then, the H&E-stained retina samples were imaged using a microscope (BX50, Olympus, Tokyo, Japan).

**FIGURE 2:**
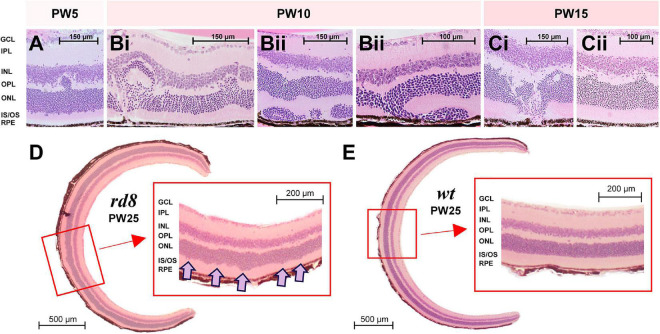
Diverse shapes of retinal folds were found in H&E staining images of *rd8* retinas at various ages. **(A)** A retinal fold was observed from a retina of a *rd8* mouse sacrificed in postnatal weeks (PW) 5. Scale bar is displayed on each panel. GCL, ganglion cell layer; IPL, inner plexiform layer; INL, inner nuclear layer; OPL, outer plexiform layer; ONL, outer nuclear layer; IS/OS, inner segment/outer segment of photoreceptor; RPE, retinal pigment epithelium. **(B)** Same as panel **(A)** but from *rd8* animals sacrificed in PW10. Retinas shown in panels **(Bi,Bii)** are from two different animals. Two panels marked as **(Bii)** show different areas of the same retina. **(C)** Same as panel **(A)** but from an *rd8* animal sacrificed in PW15. Retinas shown in panels **(Ci,Cii)** are from the same animal but two different retinas. **(D)** Cross-sectional image of the whole retina of a PW25 *rd8* mouse. (inset) A magnified view showing a wiggly borderline between ONL and IS/OS layer. Wiggly spots are marked with pink arrows. **(E)** Same as *D* but from an age-matched wild-type (*wt*) mouse (PW25). Inset shows a clear borderline between ONL and IS/OS layer.

**FIGURE 3:**
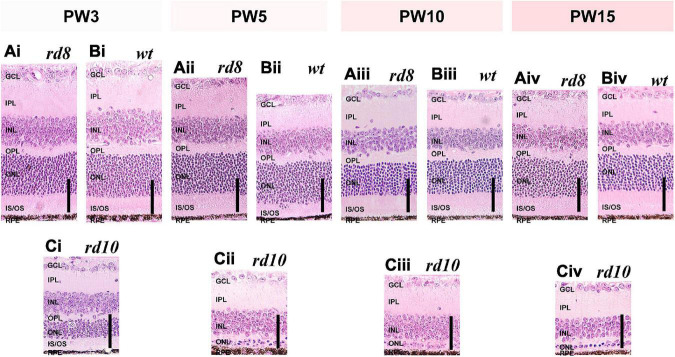
Histological analyses show similar thickness changes of retinal layers as a function of ages in *rd8* and wild-type (*wt*) retinas while *rd10* retinas show remarkably thickness decrement. **(Ai–Aiv)** H&E staining images of *rd8* retinas of mice at four age groups: postnatal weeks (PW) 3, 5, 10, and 15, respectively. **(Bi–Biv)** Same as panel **(A)** but for age-matched *wt* mouse retinas. **(Ci–Civ)** Same as panel **(A)** but for age-matched *rd10* mouse retinas. Profound thinning is observed in both ONL and IS/OS layer. Each vertical scale bar at bottom right of every panel indicates 50 μm. GCL: ganglion cell layer, IPL: inner plexiform layer, INL: inner nuclear layer, OPL: outer plexiform layer, ONL: outer nuclear layer, IS/OS: inner segment/outer segment of photoreceptor, RPE: retinal pigment epithelium.

In this study, to compare histological changes in the three different mouse models (i.e., *wt*, *rd8*, and *rd10* mice; Figures 2, 3) as a function of the aging/degeneration level, animals were sacrificed at various ages ranging from 3 to 25 weeks old. In the case of *rd10* mice, it has been known that almost no visual responses are recordable after postnatal days (PD) 60, indicating the advanced stage of retinal degeneration (Gargini et al., 2007). According to previous literature (Aredo et al., 2015), *rd8* animals at postnatal weeks (PW) 3–25 were thought to be at the early stage of retinal degeneration based on their phenotypes such as yellow fundus spots caused by subretinal microglia/macrophages. Also, in the aspects of retinal outer nuclear layer (ONL) thickness (Figure 3) and electroretinogram (ERG) signal loss (Table 1), the *rd8* mice used in this work can be considered at the early degeneration stage because they were similar to the early stage of *rd10* animals (Hawes et al., 2000; Chang et al., 2002).

### 2.3. Preparation of retina and electrophysiology

For electrophysiological recordings, all retina tissues of *rd8* (PW8–18) and *wt* (PW8–10) were isolated from an eyeball after the euthanasia and flat-mounted on a filter paper with the photoreceptor layer facing down. The prepared sample was immersed in a customized chamber attached on a slide glass. Cell-attached patch clamping method was used to record spiking activities of alpha retinal ganglion cells (RGCs) in both *wt* and *rd8* mice. Alpha RGCs were identified by their large (>20 μm) somata (Pang et al., 2003; Murphy and Rieke, 2006). Preparation of retinal sample and physiological/electrophysiology recording were conducted under the red illumination.

Glass pipettes were tailored using a micropipette puller (Model P-97, Sutter Instrument, Novato, CA, USA) and used as patch electrodes (8–12 MΩ). Two chloride-coated silver wires shaped in balls were placed at the two opposite edges of the recording chamber to serve as ground electrodes. Data were recorded and low-pass filtered at 2 kHz using an amplifier (MultiClamp 700B, Molecular Devices, Sunnyvale, CA, USA). Acquired data were digitized by a data acquisition card (PCI-MIO-16E-4, National Instruments, Austin, TX, USA). During the recordings, oxygenated Ames’ medium (Sigma-Aldrich, St. Louis, MO, USA) was continuously perfused at ∼4 ml/min and the temperature was maintained at 34–36°C. There was a small hole (∼2 mm in diameter) at the center of the filter paper that allowed light stimulation from the bottom of the microscope stage.

### 2.4. Light stimulation for RGC type classification

**FIGURE 4:**
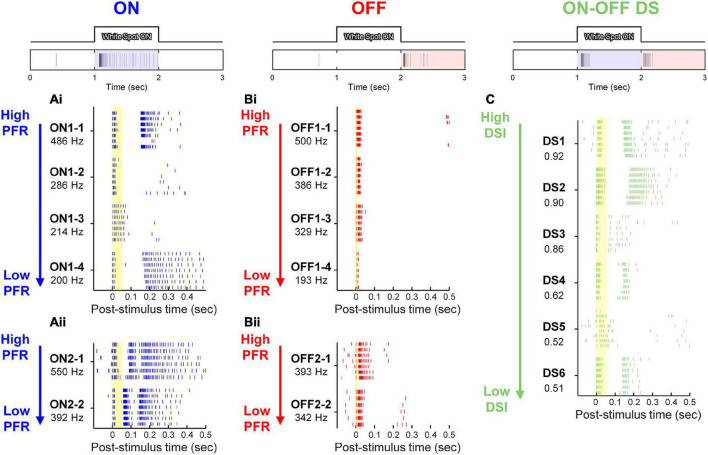
Electrically-evoked responses from 6 representative RGCs in the three physiological types of *rd8* retinas. At top of each panel, representative light-evoked responses of ON, OFF, and ON-OFF DS RGC to 1-s-long white spot flash are shown. **(Ai,Aii)** Raster plots of ON RGCs are shown in the order of peak firing rate (PFR), which is shown below each cell ID. Each vertical tick of raster plots indicates a single spike. Each cell contains responses to stimuli repeated for 6 or 7 times. Spiking patterns of ON RGCs were divided into either two **(Ai)** or three **(Aii)** bursts. **(Bi,Bii)** Same as panel **(A)** but for OFF RGCs. The responses of OFF cells were also divided into two types: abrupt spiking ending **(Bi)**, and gradual tapering off of spiking activity **(Bii)**. **(C)** Raster plots of ON-OFF DS RGCs are displayed in the descending order of direction selectivity index (DSI). Yellow vertical bands in raster plots indicate the range of early response of each cell type (i.e., 0–50, 0–6, and 0–55 ms for ON, OFF, and DS RGCs, respectively).

Physiological types of RGCs were identified by following two steps: First, a white spot on a gray background was projected onto the photoreceptor layer of target cells. Depending on their responses to the 1-s-long stationery spot flashes (diameter ranged from 100 to 1,000 μm and the biggest responses were used for later correlation analyses), RGCs were classified into either ON, OFF, or ON-OFF types (Figure 4). Second, we additionally identified whether the recorded cells were direction-selective (DS): a long white rectangular bar (300 μm × 1,800 μm) on a gray background was moved in 12 different directions (0–330° in 30° steps) at 600 μm/s. ON-OFF DS RGCs showed consistent spiking responses to both leading and trailing edges of the bright moving bar, which were ON and OFF responses, respectively. Throughout the present study, DS RGCs exclusively mean ON-OFF DS RGCs because we excluded ON type of DS cells in our analyses (Im and Fried, 2016a).

In summary, this work analyzed responses of the non-DS ON, non-DS OFF (hereafter referred to simply as ON and OFF, respectively), and ON-OFF DS RGCs in *rd8* and *wt* retinas (*n* = 14, 15, and 6 for ON, OFF, and DS cells from 19 different *rd8* retinas; *n* = 10 and 11 for ON and OFF cells from 14 *wt* retinas). The light stimuli were delivered to the retina sample using an LCD projector (PH550, LG, Seoul, South Korea) and every light stimulus was repeated at least three times for a given cell.

### 2.5. Electric stimulation

Electrical stimuli were delivered by a 10 kΩ platinum-iridium electrode (MicroProbes, Gaithersburg, MD, USA); its conical tip had a height of ∼125 μm and a base diameter of ∼30 μm. The top portion of the electrode was exposed with no insulation layer, which had a surface area of ∼5,900 μm^2^. After touching the inner limiting membrane (ILM), the tip of the stimulating electrode was positioned ∼25 μm above the ILM surface and ∼50 μm laterally away from the target cell body using a micromanipulator (MPC-200, Sutter Instrument, Novato, CA, USA). The electric stimuli were generated by a stimulus generator (STG2004, Multi-Channel Systems GmbH, Reutlingen, Germany). A monophasic cathodal current of 100 μA in amplitude (i.e., −100 μA) was delivered for 4 ms. An identical electric stimulus was repeated typically seven times (at least six times) to a given cell. Data acquisition and electric stimuli were controlled by custom software written in LabVIEW (National Instruments, Austin, TX, USA) and MATLAB (MathWorks, Natick, MA, USA).

### 2.6. Analyses of RGC spiking responses

**FIGURE 5:**
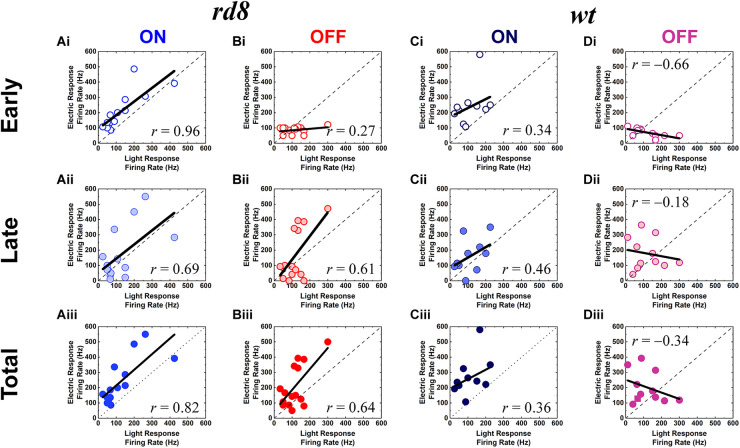
Electric responses are well correlated with light responses in both ON and OFF RGCs of the *rd8* retinas, and ON but not OFF RGCs of the *wt* retinas. **(Ai–Aiii)** Scatter plots of peak firing rate (PFR) for electric response vs. PFR for light response of the ON RGCs in the *rd8* retinas. Scatter plots are shown for **(Ai)** early, **(Aii)** late, and **(Aiii)** total response, respectively. Each data point is from a different cell. Dashed line indicates linear fitting curve of all data points, and the level of correlation (*r*-value) is shown in each plot. **(Bi–Biii)** Same as panels **(Ai–Aiii)** but for the OFF RGCs in the *rd8* retinas. **(Ci–Ciii)** Same as panels **(Ai–Aiii)** but for the wild-type (*wt*) mouse retinas. **(Di–Diii)** Same as panels **(Bi–Biii)** but for the *wt* mouse retinas.

**FIGURE 6:**
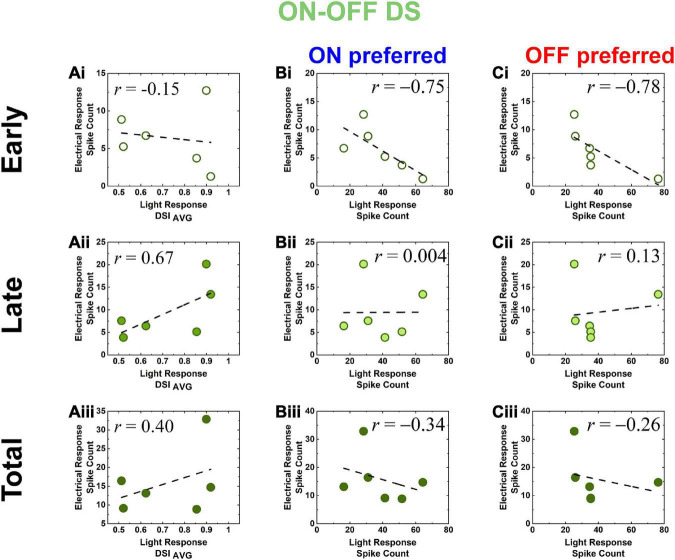
Electric response magnitudes (spike count) of DS RGCs in *rd8* and *wt* retinas show opposite correlations with their direction selectivity indices and light response spike count. **(Ai–Aiii)** Scatter plots of electric response [early, late, and total responses in panels **(Ai–Aiii)**, respectively] spike count vs. light response DSI_*AVG*_ in the same cell for all DS RGCs recorded from *rd8* retinas. Each data point is from a different cell. Level of correlation (*r*–value) is shown in each plot. Dashed line indicates linear fitting curve in each panel. **(Bi–Biii)** Scatter plots of electric response (spike count) vs. leading edge (ON) of moving bar light response (spike count) in the same cell for all DS RGCs recorded from the r*d8* retinas. Scatter plots are shown for **(Bi)** early, **(Bii)** late, and **(Biii)** total responses, respectively. **(Ci–Ciii)** Same as panels **(Ai–Aiii)** but for trailing edge (OFF) of moving bar light response.

Timings of stimulus-evoked spikes were detected by custom MATLAB code. In the case of electrically-evoked spikes, additional code was used before the spike detection to remove electric artifacts from raw recordings. Also, the spiking activities of electric responses were divided into early, late, and total responses in the same way of previous studies (Tsai et al., 2009; Im and Fried, 2015, Im et al., 2018; Lee and Im, 2018, 2019; Yoon et al., 2020; Otgondemberel et al., 2021). In other words, to divide direct and indirect responses, we separate the spiking activities of each type RGC into early and late responses based on the end of first burst (Figures 4A–C). The range of early response was displayed on raster plots (Figures 4A–C) of each RGC type with yellow bands (i.e., spikes elicited within 50, 6, and 55 ms from the stimulus onset for ON, OFF, and DS RGCs, respectively) (Yoon et al., 2020; Otgondemberel et al., 2021). The rest of the spiking activities was referred to as late responses. These ranges for early and late responses were largely similar in responses to electric pulses ranging from several hundred microseconds to Roh et al. (2022) and several milliseconds (Im et al., 2018). To investigate correlations between response magnitudes of light- and electrically-evoked spiking activities, we created scatter plots for peak firing rate (PFR) or spike count for each component of electric responses (i.e., early, late, and total response) (Figures 5, 6). We computed firing rates in each 20-ms-long bin with a rolling step of 5 ms (Figure 5).

To further characterize the DS cell responses, we calculated direction selectivity indices (DSIs) from the light responses arising from the moving bars as outlined in our previous work (Im and Fried, 2016a; Otgondemberel et al., 2021) by using the following equation:


$$\text{DSI}=1-\frac{{\text{Area}}_{\text{Preferred}}}{{\text{Area}}_{\text{Null}}}$$

where Area_*Preferred*_ and Area_*Null*_ are the areas of the preferred- and the null-side halves in the polar plots of their moving bar responses (Im and Fried, 2016a). The preferred direction was first determined as the vector sum of spiking responses arising from the white bars moved in all 12 directions, and then the null direction was assigned to be the opposite to the preferred direction. The DSI_*ON*_ and DSI_*OFF*_ were computed from polar plots of ON and OFF responses, then averaged for DSI_*AVG*_ which was used in the scatter plot (Figure 6). We excluded RGCs which had DSI_*AVG*_ < 0.5 to limit our study for highly directional cells.

**FIGURE 7:**
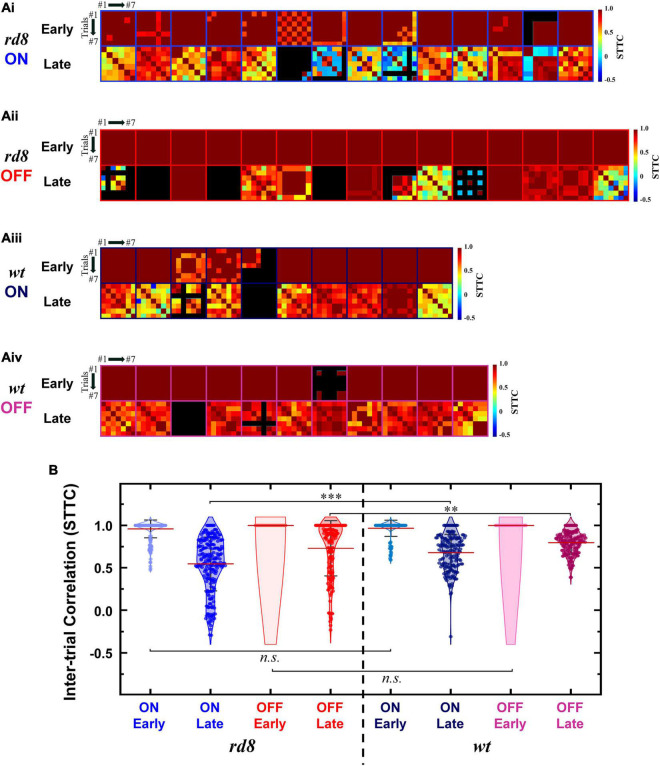
Spike timing of late response becomes less consistent in both ON and OFF RGCs of *rd8* than wild-type (*wt*) retinas. **(Ai)** Color-coded heatmaps of the spike time tiling coefficients (STTCs) of early and late responses for each ON RGC from the *rd8* retinas. **(Aii)** Same as panel **(Ai)** but for OFF RGCs in the *rd8* retinas. **(Aiii,Aiv)** Same as panels **(Ai,Aii)** but for the *wt* mouse retinas. An identical stimulus repeated typically for 7 times (at least 6 times). Black color in matrices indicates no response was elicited in those trials. **(B)** Violin plots of all STTCs computed from all *rd8* and *wt* RGCs. Red horizontal line indicates average STTC value of each group. Four violin plots on the left side are from RGCs of the *rd8* retinas and the other four violin plots on the right side are from the *wt* retinas. Statistical significance was assessed using the one-way ANOVA with Holm-Sidak post-hoc comparisons; ^***^p < 0.001, ^**^p < 0.01, and *n.s.* means not significant.

**FIGURE 8:**
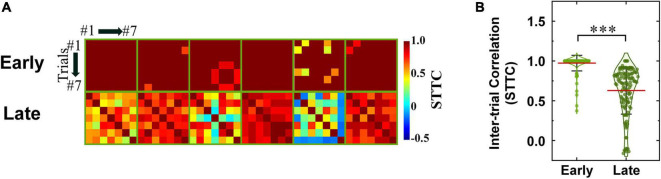
Spike timing consistencies of DS RGCs in *rd8* retinas are comparable to those of DS RGCs in *wt* retinas. **(A)** Color-coded heatmaps of the spike time tiling coefficients **(STTCs)** of early and late responses in the *rd8* DS RGCs. An identical stimulus repeated typically for seven times (at least six times). Black color in matrices indicates no response was elicited in those trials. **(B)** Violin plots of all STTCs computed from *rd8* RGCs. Red horizontal line indicates average STTC value of each group. Statistical significance test was performed using the one-way ANOVA with Holm–Sidak *post-hoc* comparisons; ^***^p < 0.001. Statistical significance comparisons between *rd8* and *wt* RGCs are shown in Table 2.

We also examined the spike timing consistency of electric responses across repeated stimulation (typically 7 trials and at least 6 trials) by computing the spike time tiling coefficient (STTC) which is defined by the following equation (Cutts and Eglen, 2014):


$$\text{STTC}=\frac{1}{2}\,\left(\frac{P_{A}-T_{B}}{1-P_{A}\,T_{B}}+\frac{P_{B}-T_{A}}{1-P_{B}\,T_{A}}\right)$$

where *P*_*A*_ is the proportion of spikes from spike train *A* that lie within time window (±Δ*t*) of each spike from spike train *B*, *T*_*A*_ is the proportion of the total recording period which contains any spikes within ± Δ*t* from spike train *A*. *P*_*B*_ and *T*_*B*_ are similarly calculated. In the present work, we used Δ*t* of 10 ms for the STTC computation. Inter-trial pair-wise STTC values were visualized as heatmaps for early and late responses (Figures 7A, 8A, for non-DS and DS RGCs respectively). Every STTC values were also shown as violin plots (Figures 7B, 8B).

### 2.7. Statistical analysis

To evaluate the correlation level between light and electric responses (Im and Fried, 2015), we computed Pearson’s product-moment coefficient (hereafter referred to as Pearson’s *r* or *r*-value) from data points shown in each scatter plot (e.g., PFRs and spike counts of electric vs. light responses). Statistical comparisons were performed using one-way ANOVA with Holm-Sidak post-hoc analyses to examine the statistical significance.

## 3. Results

### 3.1. Retinal degeneration pattern of *rd8* mice is distinct

It has been well known that phenotypes such as the speed and the pattern of retinal degeneration are substantially different depending on the mutation genes (Chang et al., 2002). For instance, although *rd1/rd10* and *rd8* mice are all RP mouse models, they showed significant distinctions in their phenotypes due to genetic differences as summarized in Table 1. The distinctive phenotypic features of the *rd8* model include large white retinal deposits and idiosyncratic retinal foldings (Chang et al., 2002; Mehalow et al., 2003). Indeed, our own H&E staining images showed retinal foldings of the *rd8* mice at various ages (Figures 2A–C). To longitudinally study morphological changes as a function of the degree of retinal degeneration, we sacrificed *rd8* mice at postnatal weeks (PW) 3, 5, 10, 15, and 25. Both size and shape of retinal foldings varied depending on neither age (at least in the age range we tested) nor locations (e.g., central vs. peripheral retinas) (Figures 2A–C). For example, older animals did not necessarily show bigger retinal foldings, consistent with a previous report (Mehalow et al., 2003). Also, even in the oldest animal we tested (PW25), there were retinal slices that had no retinal foldings observed in the whole eyeball cross-sections (Figure 2D). There was no bias of the retinal folding spots to either central or peripheral retinas. Intriguingly however, its outer retinal border of the outer nuclear layer (ONL; photoreceptor cell body layer) was wiggly as clearly shown in the inset (*see* pink arrows of Figure 2D). This wiggly borderline was found in most slices at various portions throughout all age ranges (i.e., PW3-25) of *rd8* mice we used for H&E staining, which was also reported in the previous work (Mehalow et al., 2003). Probably, the severely wiggling spots at the early onset of degeneration may further develop to be retinal foldings in *rd8* mice. In contrast, the PW25 *wt* retina showed the straight border of the ONL (Figure 2E).

We also compared histological changes of non-folding/-wiggly areas of the *rd8* retinas with age-matched *wt* and *rd10* samples. The ONL thickness change with aging of the *rd8* mice was similar to that of *wt* mice until PW15 (*compare* Figures 3A vs. 3B), suggesting the photoreceptor degeneration was minimal in the non-folding/-wiggly areas. In detail, the ONL thicknesses of the *rd8* and *wt* retinas at PW3 were ∼57 and ∼62 μm, respectively (Figures 3Ai, 3Bi), which were at the time point where retinal development was just completed. Then, the ONL thicknesses remained quite similar until PW5 (Figures 3Aii, Bii). The ONLs were shown to be thinning down from PW10 (Figures 3Aiii, Biii), and the thicknesses reached down to ∼55 and ∼51 μm at the age of PW15 (Figures 3Aiv, Biv). In contrast, the ONL thickness in *rd10* mice at PW3 was only ∼28 μm (Figure 3Ci), which is about half of the thickness of *wt* and *rd8* mice at the same age. The *rd10* ONL became only 2–3 rows of nuclei at PW5 (Figure 3Cii) and finally showed a single row of nuclei in the thickness of ∼8 μm at PW15 (Figure 3Civ). Taken together, these results clearly show much slower degeneration speed of the *rd8* mice than the *rd10* counterpart. Therefore, the *rd8* model offers possibilities of not only studying the other genotype but also carefully exploring the early stage of retinal degeneration.

### 3.2. Electrically-evoked response patterns of individual *rd8* RGCs seem largely similar to those of wt RGCs but their populational characteristics differ

Although *rd8* mice have been used for some histological studies (Chang et al., 2002; Mehalow et al., 2003; Hippert et al., 2015), it has not been explored how their RGCs respond to electric stimulation. Given the minimal retinal thinning (Figure 3) and the sporadic retinal foldings (Figure 2), there is a likelihood that electrically-evoked responses of *rd8* RGCs may have similar spiking features with those of *wt* RGCs. On the other hand, the retinal degeneration may differentially alter complex retinal circuitries of distinct physiological types of RGCs. Accordingly, we questioned how similar spiking activities arise in each RGC type between the *rd8* and *wt* retinas. To compare with our previous results recorded from *wt* and *rd10* mice (Yoon et al., 2020; Otgondemberel et al., 2021), the *rd8* retinas were electrically stimulated with a 4-ms-long monophasic cathodal current (−100 μA). We recorded spiking responses from 14 ON, 15 OFF, and 6 DS RGCs of *rd8* animals at PW8-18, and analyzed the RGCs as a whole regardless of the ages because the retinal foldings did not show dependence on age (Figure 2) and the histological changes (i.e., thickness of ONL) of the *rd8* retinas were minimal during that time period (Figure 3). A previous work also indicated the relatively steady state in ONL thickness of the *rd8* retinas between PW3 and PW12 as compared to other mouse models including *rd1* mouse (Hippert et al., 2015).

In the early degeneration state, the light-evoked spiking activities of the *rd8* retina were still strong enough to distinguish the RGC type (top row of Figure 4). Among all RGCs classified by their light responses, the electrically-evoked responses of representative 6 cells of each RGC type were shown (Figures 4A–C), which showed the highest peak firing rate (PFR) in ON and OFF types (Figures 4A, B). The spiking patterns of those RGCs seemed largely similar to those reported earlier by our group from the healthy retinas (Tsai et al., 2009; Im and Fried, 2015, 2016b; Im et al., 2018; Lee and Im, 2019; Otgondemberel et al., 2021), but minor deviations were observed in responses of ON and DS RGCs. For example, most ON RGCs showed two bursts of spikes which were separated by silent periods (*n* = 12/14; Figure 4Ai), while some ON RGCs showed three bursts (*n* = 2/14; Figure 4Aii). These results were similar to the spiking patterns of the ON brisk sustained (BS) and brisk transient (BT) sub-types of the rabbit retinas, respectively; however, the mouse alpha RGCs we targeted are known to have sustained type only in the ON pathway. In our previous recordings from the *wt* mouse retinas, all ON alpha RGCs generated two bursts of spikes (Lee and Im, 2018, 2019). Thus, the occurrence of the ON RGCs responding with the three bursts seems to be resulted from retinal degeneration.

In contrast to the responses of ON cells, responses of OFF RGCs displayed much shorter or almost no spike-free intervals between bursts of spikes (Figure 4B), which is consistent with our earlier reports (Im and Fried, 2015, 2016b). Spiking activities of some OFF cells ended their spiking rather abruptly (*n* = 10/15; Figure 4Bi) while other OFF cells were gradually tapered off with increasing inter-spike intervals (*n* = 5/15; the first row of Figure 4Bii). These results are consistent with those observed from responses of OFF BT and BS subtypes, respectively (Im and Fried, 2015). Taken together with the emergence of the three-burst spiking ON cells, our results suggest that the early-stage degeneration of *rd8* mice may minimally affect the ON system only and more than the OFF counterpart at the individual RGC level.

The rater plots of *rd8* DS RGCs were listed in the descending order of their direction selectivity indices (DSIs) (Figure 4C) because our previous research that used *wt* mouse retinas found electrically-evoked late responses lasted longer when DSI was smaller (Otgondemberel et al., 2021). However, the DS RGCs of *rd8* animals showed the opposite trend: the late responses (i.e., spiking activities outside of the yellow band indicating early responses in Figure 4C) were usually longer lasting with bigger DSI (except DS3 and DS5). This contrast was another minor deviation between the *rd8* and *wt* retinas, raising a possibility that populational response characteristics of DS RGCs may be different between the two groups.

### 3.3. Correlations between response magnitudes to electric vs. light stimuli were differently altered depending on pathways in *rd8* mice

To quantitatively analyze populational response difference between the *rd8* and *wt* retinas in non-DS RGCs, we plotted the PFR of electric vs. light responses of ON and OFF RGCs (Figure 5). In those scatter plots, the correlation levels between the two responses were characterized to see if how similar spiking response magnitudes can arise from electric stimulation, which seems critical for high-quality prosthetic vision that can be better perceived (Im and Fried, 2015). Interestingly, for all three components (e.g., early, late, and total responses) of electric responses in comparison with their own light responses, the ON/OFF RGCs of the *rd8* retinas showed similar/opposite correlation levels with those of *wt* retinas, respectively (*compare* Figures 5A vs. 5C for similar correlation levels in ON RGCs, and Figures 5B vs. 5D for opposite correlation levels in OFF RGCs).

From the prosthetic perspective, it is particularly notable that both ON and OFF RGCs in the *rd8* retinas generated electric responses which were positively proportional to light responses in terms of spiking magnitudes (Figures 5A, B). For example, the PFRs of the total responses of the *rd8* retinas had fairly high *r*-values for both ON and OFF RGCs (0.82 and 0.64 in Figures 5Aiii, Biii, respectively). Although the positive correlation between the two responses seems to be preferred for appropriate perception of electrically-evoked artificial neural signals, the correlation difference between the ON and OFF pathways may be essential for prosthetic users to discern luminance increment/decrement at a given location of the visual space (Im and Fried, 2015). Because the indiscriminate activations of ON and OFF RGCs which tile the retina at different stratification depths are inevitable with currently available electric stimulation methods, it may induce difficulties in determining whether the stimulation spot is bright or dark if electric responses of both ON and OFF channels are well-correlated (i.e., similar) with their light responses (see section “Discussion”). In contrast to the *rd8* cells, the RGCs of *wt* retinas demonstrated contrary correlation levels between electric vs. light responses in the ON vs. the OFF systems: the ON RGCs showed the positive correlations (*r* = 0.34, 0.46, and 0.36 in early, late, and total response; Figures 5Ci–Ciii) while the OFF cells exhibited negative correlations (*r* = −0.66, −0.18, and −0.34 in early, late, and total response, respectively; Figures 5Di–Diii). These contrasting correlation levels between ON and OFF pathways in the *wt* mouse retinas are consistent with those reported from the healthy rabbit retinas (Im and Fried, 2015). Also, this contrast is expected to make electric responses of ON cells better perceivable than those of OFF cells (Im and Fried, 2015), probably eliciting “*bright*” phosphenes in prosthetic users. It is noteworthy again, however, that the *rd8* RGCs did not generate the opposite correlation levels between the ON and the OFF types (*compare* Figures 5A, B).

Another distinct populational features of the *rd8* RGCs was also observed in DS RGCs: since the responses of *wt* DS RGC were already investigated by creating scatter plots of spike counts in our earlier work (Otgondemberel et al., 2021), we similarly plotted spike counts as a function of average DSI (DSI_*AVG*_) (Figure 6A). Spike counts of the early responses in the *rd8* DS RGCs yielded a weak negative correlation with the DSI_*AVG*_, which was somewhat analogous to *wt* ones (*r* = −0.15 vs. −0.57 for *rd8* vs. *wt*; see Otgondemberel et al., 2021 for *wt* data). However, spike counts of the late and the total responses showed sharp contrasts between the *rd8* and *wt* retinas, having positive correlations in the *rd8* DS RGCs (*r* = 0.67 and 0.40 for the late and the total responses, respectively; Figures 6Aii, Aiii) but negative correlations in the *wt* DS RGCs (*r* = −0.98 and −0.90 for the late and the total responses, respectively; see Otgondemberel et al., 2021 for *wt* data). These contrasting results suggest that the activation of inhibitory presynaptic neurons of the retinal circuit might be fairly decreased even from the early stage of degeneration (see section “Discussion”). Taken all together, although it was hard to notice any substantial difference in spiking patterns of individual RGCs in the *rd8* retinas (minimal difference in ON RGCs and almost no difference in OFF and DS RGCs; see Figures 4B, C) compared to those of the *wt* retinas (Lee and Im, 2018, 2019; Yoon et al., 2020; Otgondemberel et al., 2021), their populational characteristics appeared to be markedly altered in OFF and DS pathways (Figures 5, 6A) even with the early progression of retinal degeneration caused by *Crb1* mutation.

Since the generation of robust spiking responses to both increment and decrement of luminance is another hallmark of DS RGCs, it is important to know, for retinal prosthetic application, if one component of light responses (i.e., ON or OFF) correlates better with its electric response over one another. In scatter plots of the DS RGCs (Figures 6B, C), the light and electric responses generally showed negative correlations in the *rd8* retinas (early and total electric responses with ON and OFF preferred light responses; Figures 6Bi, Biii, Ci, Ciii). Meanwhile, the late electric response of the *rd8* DS cells showed little or almost no correlation with either ON or OFF light responses (*r* = 0.004 and 0.13 for ON and OFF preferred, respectively; Figures 6Bii, Cii). In the case of *wt* retinas (Otgondemberel et al., 2021), however, the spike count correlation between light and the electric responses showed positive correlations in all response components (i.e., early, late, and total responses). It is noteworthy that the overall tendencies in the correlations between electric vs. light responses were opposite between the *rd8* vs. *wt* DS cells. Given the young ages (PW8-18) of the *rd8* animals, our results suggest that the complex retinal circuit of DS cells may be affected even from the early-stage retinal degeneration. Accordingly, it would be intriguing to study how the complicatedly-functioning RGC types respond differently to electric stimulation at the early stage of RPs and how they lead to different clinical outcomes of retinal prostheses.

### 3.4. Trial-to-trial spiking consistency in response to electric stimulation was affected only in non-DS RGCs of *rd8* mice

Healthy neural systems decrease their spiking variability across trials in response to external stimulus (Churchland et al., 2010). However, neurodegeneration seems to increase spiking variabilities in neural responses to stimuli: we recently reported that the variabilities of *rd10* RGC spiking activities arising from electric stimulation increased with the advancing level of the retinal degeneration (Yoon et al., 2020). This increased inconsistency is likely to prevent electrically-evoked spiking activities from being accurately perceived by the higher visual centers (Yoon et al., 2020). In the case of RGCs in the *rd8* retinas, the reduced consistency was not readily notable from the raster plots shown earlier (Figures 4A, B). However, a couple of *rd8* DS cells (i.e., DS3 and DS5) showed relatively higher variability across repeats of an identical stimulus than other DS RGCs which demonstrated consistent spiking patterns across trials (*n* = 4/6; Figure 4C).

To more systematically investigate the level of inter-trial variabilities of the *rd8* responses to electric stimulation, we calculated the spike time tiling coefficients (STTCs) and compared them with those from the *wt* mice (Figures 7, 8 for non-DS and DS RGCs, respectively). All STTC values of early and late responses across 6–7 repeated stimuli were plotted as color-coded heat matrices for individual cells (Figures 7A, 8A) and violin plots for each type (Figures 7B, 8B). Commonly, regardless of the cell type and the strain, the STTC values of the early responses were markedly high (see first rows of Figures 7Ai–Aiv, 8A). Also, no statistical significance was found between the early responses of the *rd8* and *wt* RGCs (Figures 7B for non-DS cell; *rd8* DS RGC data were compared with *wt* DS RGC data reported in Otgondemberel et al. (2021). However, the spike timing variabilities of the late responses were significantly increased in the *rd8* RGCs compared to the *wt* RGCs but only in non-DS types (Figures 7B). Also, the difference in the average STTCs (STTC_*AVG*_) between the *rd8* and *wt* late responses was larger in the ON RGCs (0.55 and 0.68 from the *rd8* and *wt* retinas, respectively; *p* < 0.001; Figure 7B) than in the OFF RGCs (0.73 and 0.80 from the *rd8* and *wt* retinas, respectively; *p* < 0.01; Figure 7B). In our previous study (Yoon et al., 2020), the inter-trial consistency of late response in the *rd10* ON RGCs decreased progressively from postnatal days (PD) 15–60, whereas the STTCs of the *rd10* OFF RGCs remained unchanged until PD19 and then decreased drastically from PD31. Therefore, the response consistency-wise, the degeneration level of the *rd8* mice (PW8-18) used in this study seems to be similar to that of the *rd10* mouse at the age between PD19 and 31. Although the histological analyses showed too different retinal thicknesses (Figure 3), it is likely that the neuronal component which is critical for the spiking response consistency may undergo similar level of degeneration in those two age groups in the two strains (i.e., PW8-18 of *rd8* mice and PD19-31 of *rd10* mice).

**FIGURE 9:**
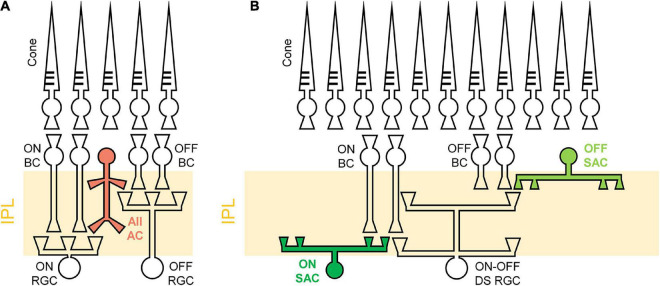
Schematic illustrations of different retinal circuits between non-DS and DS RGCs. **(A)** AII amacrine cell (AC) has a narrow field of bistratified dendrites to ON and OFF RGCs. **(B)** Starburst amacrine cells (SACs) stratify their dendrites to a relatively wide area to modulate direction-selective light responses of DS RGCs. IPL, Inner plexiform layer; BC, bipolar cell; RGC, retinal ganglion cell; AII AC, aII amacrine cell; SAC, starburst amacrine cell; DS RGC, directional selective retinal ganglion cell. Rod pathways are not illustrated for brevity.

Similarly, the spiking consistency of DS RGC was also investigated (Figure 8). Interestingly, even though the correlation levels between electric and light responses of the *rd8* DS RGCs were quite different from those of *wt* ones (Figure 6), the variabilities of spike timing were well maintained in the *rd8* retinas (Figure 8) compared to the *wt* retinas (Otgondemberel et al., 2021). Because of DS3 and DS5, the STTC_*AVG*_ of the *rd8* DS cells was slightly decreased, but it was not significantly different from that of the *wt* DS cells. Taken all together, the early-stage retinal degeneration in the *rd8* model seems to differentially affect RGC response consistencies to electric stimulation depending on the pathway (i.e., the ON pathway most and the DS pathway least). It has been well known that non-DS and DS RGCs have distinct types of presynaptic inhibitory neurons such as AII vs. starburst types of amacrine cells for non-DS and DS RGCs, respectively (Figure 9). Perhaps, those inhibitory neurons might have been affected in different degrees by retinal degeneration of the *rd8* mice, and probably causing the difference in the electrically-evoked spiking consistency changes (*see* DISCUSSION).

## 4. Discussion

### 4.1. Early retinal degeneration alters electrically-evoked response features of RGCs depending on physiological types

We first expected RGCs in the *rd8* mice would generate spiking patterns of electric responses which are similar to those of *wt* RGCs due to the following two reasons: First, the ages of the *rd8* mice used in our experiments were ranging from PW8 to 18, which can be considered to be at very early degeneration stage (Hawes et al., 2000; Chang et al., 2002; Aleman et al., 2011; Aredo et al., 2015; Hippert et al., 2015). Also, our previous report using *rd10* mice (Yoon et al., 2020) also demonstrated that electric responses of ON and OFF RGCs at early degeneration stage are not that altered in comparison with those of *wt* mice. Second, the areas affected by retinal degeneration were small as shown in a little portion of retinal slices of the *rd8* mice (Figure 2) although we were not able to quantify the exact ratio of retinal foldings or wiggly borderline because we did not investigate every single slice of the whole retina. Consistent with our initial expectation, the spiking patterns arising in the *rd8* RGCs looked quite akin to those of the *wt* RGCs in corresponding types (Figure 4). However, the populational characteristics of the electric responses were substantially different between the *rd8* and *wt* RGCs. In addition, those populational differences were distinct in each type (i.e., ON, OFF, and DS type) as summarized in Table 2. For example, in the cases of response magnitudes represented by PFR (Figure 5) of non-DS cells, ON RGCs in the *rd8* retinas maintained similar positive correlations like *wt* ON RGCs while OFF RGCs showed opposite correlation polarities compared to *wt* OFF RGCs (*see* “Response magnitude correlation with light responses” of ON and OFF RGCs in Table 2).

**TABLE 2:** Summary of populational electric response feature comparisons between *rd8* and wild-type (*wt*) retinas.

RGC types	Electric responses properties	*rd8*		*wt*		Related Figure
ON	Response magnitude correlation with light responses	*r* = 0.82		*r* = 0.36		Figure 5
	Response consistency (STTC)	Early response: No statistical significance Late response: *p* < 0.001 (lower in *rd8*)				Figure 7
OFF	Response magnitude correlation with light responses	*r* = 0.64		*r* = −0.34		Figure 5
	Response consistency (STTC)	Early response: No statistical significance Late response: *p* < 0.01 (lower in *rd8*)				Figure 7
DS	Response magnitude correlation with DSI_*AVG*_	*r* = 0.40		*r* = − 0.90		Figure 6
	Response magnitude correlation with light responses	ON preferred	*r* = −0.34	ON preferred	*r* = 0.40	Figure 6
		OFF preferred	*r* = −0.26	OFF preferred	*r* = 0.45	
	Response consistency (STTC)	Early response: No statistical significance Late response: No statistical significance				Figure 8

Data of ON-OFF DS in wt retina from Otgondemberel et al. (2021). For brevity, r-value indicating the level of correlation is shown for total response only.

The opposite response characteristics between the *rd8* and *wt* retinas were also observed in DS RGCs. We examined non-DS as well as DS cells because they have different physiological functions and morphology. ON-OFF DS RGCs process the dynamic visual features in mammalian retinas, which would be critical for the survival of small animals. In non-human primate retinas, the recursive bistratified RGCs seems to have ON-OFF direction selective response features (Detwiler et al., 2019). In contrast to non-DS which have monostratified dendrites, DS RGCs stretch their dendrites to two sublaminar in the IPL layer. Moreover, since DS cells are known to have complex retinal circuits (Famiglietti, 1992; Fried et al., 2002; Borst and Euler, 2011; Vaney et al., 2012; Mauss et al., 2017; Wei, 2018), we expected that electrical responses of DS RGCs could sensitively reflect the remodeling of the neural circuits even in an early stage of retinal degeneration. Since starburst amacrine cells (SACs) are highly likely to strongly involve in the spiking activities of DS RGCs as inhibitory presynaptic neurons, electric responses (spike count) of the *wt* mice were weaker with bigger DSI_*AVG*_ of their light responses (*r* = −0.90 in Table 2) (Otgondemberel et al., 2021). However, the DS RGCs in the *rd8* retina showed proportionally stronger electric responses as DSI_*AVG*_ increased (*r* = 0.40 in Figure 6Aiii and Table 2), suggesting the inhibitory components may be affected by the degeneration. Besides, different from the *wt* DS cells, the spike counts of electric responses arising in the *rd8* DS RGCs were not positively correlated with those of light responses to white bars moving in preferred direction (Figure 6 and *see “*Response magnitude correlation with light responses” of ON-OFF DS RGCs in Table 2). The negative correlation between the light and the electric responses was observed in the *rd8* retinas for both ON and OFF preferred responses (Figures 6B, C and Table2), which is a sharp contrast to the positive correlation between those in the *wt* retinas (Table2; Otgondemberel et al., 2021). It may be helpful for the retinal prosthetic community to thoroughly investigate RGC responses to electric stimulation as a function of degeneration levels because it can offer some insights regarding critical implantation time window for maximal clinical efficacy (Yoon et al., 2020). Also, it must be crucial to uncover the underlying mechanism(s) of our findings observed even at the early stage of retinal degeneration more successful clinical outcomes of retinal implants. For example, it seems important to identify neuronal component(s) altering the populational response features in the OFF pathway, which may require early intervention before any irreversible molecular change(s) happen over the course of retinal degeneration.

Our results may be explained by the retinal foldings caused by *Crb1* mutation, which implies remodeling of the retinal circuit, especially close to the photoreceptor and bipolar cell layers. It is well known that we can selectively stimulate ganglion cells, bipolar cells, or photoreceptors by modulating electrical stimulus duration (Fried et al., 2006; Freeman et al., 2010). In detail, ganglion cells that have voltage-gated sodium channels are rapidly activated in less than 1 ms (Fohlmeister and Miller, 1997). However, voltage-gated calcium channels are slowly activated in several milliseconds and are located at photoreceptors and synaptic terminals of bipolar cells (Protti and Llano, 1998; Thoreson, 2007; Hu et al., 2009). Since we stimulate the retinal tissue with a 4-ms-long monophasic cathodal current (−100 μA), it was sufficient stimulus duration to activate indirect responses from bipolar cells and photoreceptors. Therefore, we thought partial retinal foldings of *rd8* retinas may have resulted in the quite different indirect responses compared to healthy retinas. But, it is still unclear how those changes were not uniform across RGC types.

These differences in electric response features across RGC types may be caused by the different retinal neural circuits including types of inhibitory presynaptic neurons. For example, the AII amacrine cells (ACs) are known to stratify their dendrites in both ON and OFF sublaminae (Figure 9A), modulating responses of non-DS ON and OFF RGCs (Strettoi et al., 1992; MacNeil and Masland, 1998, MacNeil et al., 1999; Tsukamoto and Omi, 2017). For DS RGCs, the SACs are known to play a critical role in the generation of directionally-selective responses by suppressing responses to null-direction motions (Famiglietti, 1992; Fried et al., 2002; Borst and Euler, 2011; Vaney et al., 2012; Mauss et al., 2017; Wei, 2018). One notable point is that SACs have relatively wider dendritic fields than AII ACs (Figure 9B) (MacNeil et al., 1999; Lin and Masland, 2006), suggesting that it is more likely that the sporadic foldings in the *rd8* retinas affect DS circuits more than non-DS circuits. Moreover, due to the remarkable complexity in the retinal neural circuits of DS RGCs (Famiglietti, 1992; Fried et al., 2002; Borst and Euler, 2011; Vaney et al., 2012; Mauss et al., 2017; Wei, 2018), it is likely that even small remodeling of the retina creates a critical influence to not only light responses but also electrically-evoked responses. Certainly, the *rd8* and *wt* DS cells showed contrasting populational characteristics of electric responses in comparisons with both DSI_*AVG*_ and light response magnitudes (Figure 6).

### 4.2. Populational electric responses of ON and OFF RGCs may confound the brain of retinal prosthetic users with CRB1 gene mutation

In the earlier clinical trials of retinal prosthetic systems, the subjects perceived bright sensations, so called “*phosphenes*,” when electrical stimulation was applied (Humayun et al., 1996, 2003; Fujikado et al., 2007; Naycheva et al., 2012). However, it was not clear how those prosthetic users perceive electrically-evoked neural activities as the bright stimuli because the ON and OFF pathways of the retina are non-selectively activated near any electric stimulation site, which would be much different from the exclusive activation of either pathway during natural viewing (Dan et al., 1996; Marc et al., 2003; David et al., 2004; Touryan et al., 2005). In our previous study using the healthy rabbit retinas, we reported more physiological spiking patterns arose in response to identical electric stimulation in the ON pathway than the OFF counterpart (Im and Fried, 2015). Accordingly, even if electric pulses indiscriminately activate the two pathways, the downstream visual centers may better understand the electric responses from ON RGCs but misinterpret/ignore those from OFF RGCs, resulting in preferential reports of “*bright*” phosphenes. Consistent with this earlier work, the *wt* mouse retinas of the present study showed more physiological spiking magnitudes in response to electric stimulation in ON than OFF RGCs: positive/negative correlations with light responses were observed in ON/OFF RGCs, respectively (Figures 5C vs. 5D). However, in the *rd8* mouse retinas, the electric responses of both ON and OFF RGCs were positively correlated with their light responses (*compare* Figures 5A vs. 5B), indicating that populational OFF responses were similarly physiological (i.e., natural). Therefore, the electric responses of the ON and OFF channels in the *rd8* mice may confound the brain as to the luminance level (i.e., bright or dark) of the stimulation site. If that is the case, it is even more critical to explore novel stimulation strategies for increasing the response ratio between ON versus OFF responses (Tsai et al., 2009; Freeman et al., 2010; Twyford et al., 2014, Lee and Im, 2018, Lee and Im, 2019).

Given that retinal prostheses periodically deliver electric pulses, the trial-to-trial spiking consistency of individual RGCs is likely to be crucial for stable/consistent visual percepts (Yoon et al., 2020). Accordingly, it would be another downside of the *rd8* retinas that the consistencies of the late responses were significantly decreased in both ON and OFF RGCs (*p* < 0.001 and *p* < 0.01 for ON and OFF RGC, respectively; Figure 7B and Table 2), even though the consistency was not altered in DS RGCs (*see* ‘Response consistency (STTC)’ of ON-OFF DS RGC in Table 2). Since the ON and OFF pathways are known to be crucial in forming visual percepts (Im and Fried, 2015; Lee and Im, 2019), our results suggest the increased response variability of the two types of RGCs may hinder the precise perception of artificial vision. Instead of the inconsistent late responses in the degenerate retinas, it may be preferred to rely on the early responses which did not display any substantial changes in the spiking consistency. Although short electric pulses had long been thought to evoke direct spikes without late responses, it has recently been reported that late responses can be elicited with short pulses at high current amplitudes in the range of clinical use (Roh et al., 2022). Moreover, the early responses appeared less natural than the late responses (Im and Fried, 2015), remaining another challenge as to how to make overall artificial spiking activities as physiological as possible. Taken together, our results raise a possibility that retinal prosthetic users with *CRB1* gene mutation may experience more challenges to discern any bright spots or perceive somewhat sophisticated patterns.

### 4.3. The quality of prosthetic vision may vary substantially across different genotypes

Even though identical retinal prostheses were implanted, some prosthetic users did not experience any artificial vision (Stingl et al., 2015). Due to the lack of genotyping before the implantations, subjects might have various genetic mutations and resulting considerable performance variations across the users. To explore any dependence on the RP genotypes, our earlier work (Yoon et al., 2020) and the present study have thoroughly analyzed electric responses of the major RGC types in the *rd10* and *rd8* mice which have *Pde6b* and *Crb1* mutations. However, in terms of prevalence, *PDE6B* and *CRB1* genes only takes 4–5% and 1% of the population of ARRP, respectively (Hartong et al., 2006). It is also worth noting that there are still plenty genes that cause blindness (*see* Figure 1B). Thus, it is somewhat doubtful whether experimental results obtained from both *rd10* and *rd8* mouse models can be effectively translational to the whole RP population. Also, there is a possibility that different clinical outcomes of retinal prostheses (Stingl et al., 2015) might be occurred by different types of mutated genes causing RP due to its genetic diversity. Therefore, additional RP animal models with different genotypes are highly likely to contribute for the future success of not only retinal prosthetics but also other sight restoration approaches such as optogenetics. In the case of rat models which have much bigger eyeballs than mouse models, the Royal College of Surgeons (RCS) rats are most widely used, which has a mutation in *Mertk* gene (Vollrath et al., 2001). *Mertk* is the orthologue of human *MERTK* gene which is responsible for less than 1% of autosomal recessive RP (Hartong et al., 2006). To represent bigger population of RP patients, there have been recent endeavors to develop new models such as *Pde6b* gene knockout rats (Yeo et al., 2019; Yang et al., 2021).

Although it has not received much attention so far, we may need to pay extra considerations on both genotype and phenotype of patients for the further improvement of retinal implants (Kuehlewein et al., 2022). For example, although the progression of retinal degeneration seems to reduce artificial visual information (Kang et al., 2021), it may be possible that certain genotype(s) minimizes the reduction in prosthetic neural information, raising a necessity to compute the artificial visual information transmitted from RGC populations (Kim et al., 2022). Also, the field of retinal prosthetics would need fundamental neuroscience research to reveal the underlying mechanisms of our findings reported here.

### 4.4. Limitations of this study

The present study has several limitations to be considered. First, we just classified the RGCs into three physiological types according to their light responses (i.e., non-DS ON, non-DS OFF, and DS ON-OFF cells). Since the retina is a very complex and sophisticated sensory organ, which has a tremendous number of RGC types including several subtypes in both ON and OFF pathways. Previous researches reported that RGCs can be divided into more than ∼42 subtypes based on their genetic, morphological, and functional features (Boycott and Wässle, 1974; Baden et al., 2016; Kim et al., 2021; Goetz et al., 2022). Therefore, there is a possibility that some of the subtype-dependent characteristics might have beend missed in our analyses. In the future follow-up study, it seems necessary to precisely subdivide RGC types using more advanced techniques. Second, our study has not considered the effect of aging which not only changes the morphology of RGCs but also cause loss of the RGCs (Neufeld and Gachie, 2003; Bell et al., 2020; Lee et al., 2022). Moreover, there is a risk of developing glaucoma which is another representative age-related disease that damages the function of RGCs (Chrysostomou et al., 2010; Bell et al., 2020).

## 5. Conclusion

In the present work, for the first time, we have reported the distinct electric responses of RGCs in ON, OFF, and DS types from the *rd8* mice (PW8-18) which carry *Crb1* mutation, and compared those of the *wt* mice in each type. In each RGC type of the *rd8* retinas, the electrically-evoked spiking responses seemed quite similar to those arising in the corresponding type of the healthy retina. It was somehow expected since our histological analyses showed, other than sporadic retinal folds, the relatively well-maintained retinal structures, suggesting that the *rd8* mice we used in this work were at the early-stage RP. However, populational characteristics were differently altered across the RGC types in terms of the correlations between the electric vs. light response features: both DS and non-DS RGCs of the *rd8* retinas showed much distinct correlation levels/tendencies contrary to those appeared in the *wt* retinas (Table 2). Also, the consistencies of electric responses significantly decreased in both ON and OFF RGCs of the *rd8* retinas, which is one of the hallmarks of degenerate retinas compared to the *wt* retinas; but the similar reduction was not observed in DS RGCs of the *rd8* retinas. All in all, for the enhanced performance of retinal prostheses in clinical use, it seems vital to perform comparative studies of key physiological types of RGCs across animal models with various genotypes.

## Data availability statement

The original contributions presented in this study are included in the article/supplementary material, further inquiries can be directed to the corresponding author.

## Ethics statement

This animal study was reviewed and approved by Institutional Animal Care and Use Committees of the Korea Institute of Science and Technology.

## Author contributions

HR and YO conducted the cell-attached patch clamping experiments and analyzed the data. JE performed the H&E staining and summarized the histological characteristics. DK investigated the previous literatures regarding genotypes of outer retinal degenerative diseases and wrote the relevant texts. HR drafted the figures and manuscript. MI designed the study, supervised the all experiments and data analyses, and revised the figures and manuscript. All authors reviewed and approved the final manuscript before submission.
