# Corrigendum: Electrically-evoked responses for retinal prostheses are differentially altered depending on ganglion cell types in outer retinal neurodegeneration caused by *Crb1* gene mutation

**DOI:** 10.3389/fncel.2024.1397787

**Published:** 2024-04-03

**Authors:** Hyeonhee Roh, Yanjinsuren Otgondemberel, Jeonghyeon Eom, Daniel Kim, Maesoon Im

**Affiliations:** ^1^Brain Science Institute, Korea Institute of Science and Technology, Seoul, Republic of Korea; ^2^School of Electrical Engineering, Korea University, Seoul, Republic of Korea; ^3^School of Electrical Engineering, Kookmin University, Seoul, Republic of Korea; ^4^Department of Biomedical Sciences, Seoul National University College of Medicine, Seoul, Republic of Korea; ^5^Division of Bio-Medical Science & Technology, KIST School, University of Science and Technology, Seoul, Republic of Korea

**Keywords:** retinitis pigmentosa, retinal degeneration, artificial vision, retinal prosthesis, electrical stimulation

In the published article there was an error in Figure 5. The title of every *X*-axis in Figure 5 was written incorrectly as “Light Response Firing Rage (Hz).” The correct title of every *X*-axis in Figure 5 should be “Light Response Firing Rate (Hz).” The corrected Figure 5 appears below:

**Figure 5:**
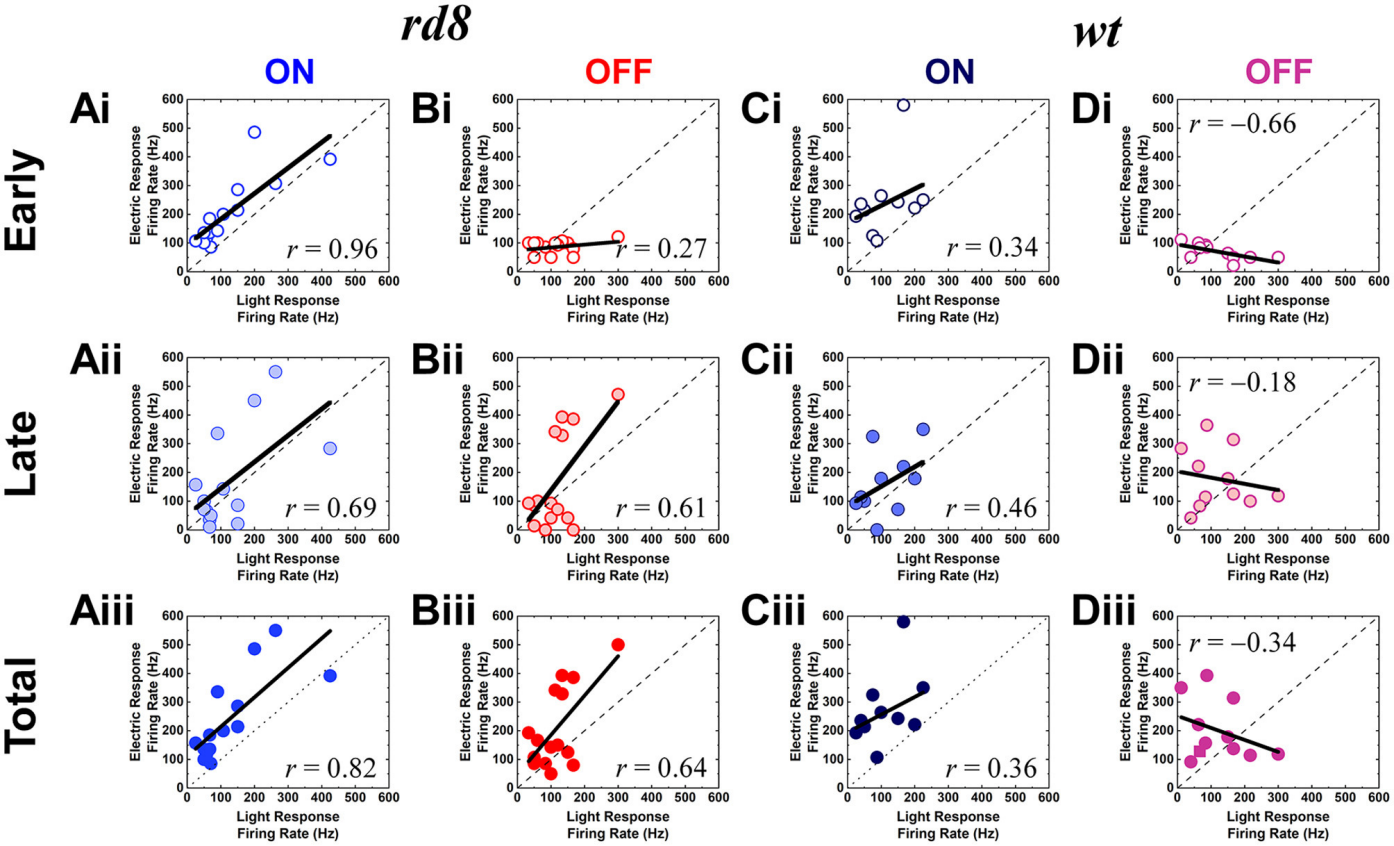
Electric responses are well correlated with light responses in both ON and OFF RGCs of the *rd8* retinas, and ON but not OFF RGCs of the *wt* retinas. **(Ai–Aiii)** Scatter plots of peak firing rate (PFR) for electric response vs. PFR for light response of the ON RGCs in the *rd8* retinas. Scatter plots are shown for **(Ai)** early, **(Aii)** late, and **(Aiii)** total response, respectively. Each data point is from a different cell. Dashed line indicates linear fitting curve of all data points, and the level of correlation (*r*-value) is shown in each plot. **(Bi–Biii)** Same as panels **(Ai–Aiii)** but for the OFF RGCs in the *rd8* retinas. **(Ci–Ciii)** Same as panels **(Ai–Aiii)** but for the wild-type (*wt*) mouse retinas. **(Di–Diii)** Same as panels **(Bi–Biii)** but for the *wt* mouse retinas.

The authors apologize for this error and state that this does not change the scientific conclusions of the article in any way. The original article has been updated.

